# Risk of symptomatic severe acute respiratory syndrome coronavirus 2 infection not associated with influenza vaccination in the 2019–2020 season

**DOI:** 10.1111/irv.12880

**Published:** 2021-06-25

**Authors:** Jennifer P. King, Huong Q. McLean, Edward A. Belongia

**Affiliations:** ^1^ Center for Clinical Epidemiology & Population Health Marshfield Clinic Research Institute Marshfield Wisconsin USA

**Keywords:** COVID‐19, influenza vaccine, outpatient, PCR, SARS‐CoV‐2

## Abstract

The association of influenza vaccine and severe acute respiratory syndrome coronavirus 2 (SARS‐CoV‐2) infection was assessed by test‐negative design using data collected for a study of outpatient COVID‐19‐like illness with onset dates from June to September 2020. Multivariable logistic regression models examined the association between receipt of 2019–2020 influenza vaccine and PCR‐confirmed SARS‐CoV‐2 with adjustment for potential confounders. Receipt of influenza vaccine during the 2019–2020 influenza season was not associated with increased odds of SARS‐CoV‐2 infection in adults (aOR 0.83, 95% CI 0.63 to 1.10) or children (aOR 0.92, 95% CI 0.47 to 1.80).

## 1 BACKGROUND

Influenza and severe acute respiratory syndrome coronavirus 2 (SARS‐CoV‐2) are respiratory viruses that cause severe illness in susceptible individuals. For influenza viruses, glycoproteins hemagglutinin (H) and neuraminidase (N) are the dominant immune epitopes. For SARS‐CoV‐2, the immune response is directed primarily against the spike (S) surface protein. Influenza viruses and coronaviruses are antigenically distinct, and there is no apparent cross‐reactivity in the immune response.^1^

However, a study published in early 2020 at the beginning of the coronavirus disease 2019 (COVID‐19) pandemic suggested a positive association (increased risk) between influenza vaccination and subsequent detection of seasonal coronavirus (human coronaviruses NL63, 229E, OC43, and HKU1) in military personnel with respiratory illness.^2^ Methodological flaws were identified that biased the results toward a positive association.^3^ The author subsequently published a letter confirming that this study provided no evidence for a link between influenza vaccination and COVID‐19 risk.^4^ However, anti‐vaccine advocates continue to raise concerns that influenza vaccination may increase the risk of COVID‐19.

Subsequent studies have failed to confirm a positive association between influenza vaccination and SARS‐CoV‐2 infection. A recent systematic review assessed the relationship between influenza vaccination and COVID‐19 in seven studies.^5^ One study was a COVID‐19 prediction model that was not intended to estimate the independent risk from influenza vaccination.^6^ The other studies found either no association or a modest reduction in risk of SARS‐CoV‐2 infection among influenza vaccine recipients after adjusting for potential confounders. A subsequent cross‐sectional analysis of patients tested for SARS‐CoV‐2 in Israel found that influenza vaccination in 2019–2020, 2018–2019, or both seasons was associated with reduced risk of SARS‐CoV‐2 infection.^7^

The test‐negative design (TND) is a robust method for estimating influenza vaccine effectiveness (VE), and yields a valid estimate of VE under most scenarios.^8^ Prospective studies of influenza vaccination and coronaviruses using the TND have been reported from Europe and Canada, but not the United States.^3^, ^9^ Further, few evaluations of influenza vaccination and COVID‐19 have examined risk in children, leaving them an understudied population. The objective of this study was to determine if receipt of 2019–2020 seasonal influenza vaccine was independently associated with laboratory‐confirmed, symptomatic SARS‐CoV‐2 infection among a population of adults and children seeking outpatient care for COVID‐19‐like illness in central Wisconsin.

## 2 METHODS

This was a secondary analysis of data from a COVID‐19 epidemiology study. Patients with an acute illness were prospectively enrolled with verbal consent. All participants had results from clinically ordered reverse transcription polymerase chain reaction (RT‐PCR) testing for SARS‐CoV‐2. To ensure complete and accurate immunization records, we restricted analyses to participants with at least two clinical encounters with a health system provider during the 36 months prior to study enrollment and any prior vaccine record in the local immunization registry (RECIN). RECIN exchanges data with the Wisconsin Immunization Registry and has been previously validated for nearly complete capture of influenza vaccinations.^10^

Patients with an acute illness ≤10 days duration (onset to swab) were eligible if they met symptom criteria which included (1) loss of taste or smell or (2) cough or fever and at least one additional respiratory (shortness of breath, runny nose/nasal congestion, and sore throat) or gastrointestinal (abdominal pain, diarrhea, and vomiting) symptom. Participants with illness onset between June 1 and September 30, 2020 were included. We excluded participants who received 2019–2020 influenza vaccination <14 days prior to illness onset and participants who received 2020–2021 influenza vaccine any time prior to illness onset.

We assessed the association between influenza vaccination and COVID‐19 using the TND. A case was defined as a study participant with a positive RT‐PCR result for SARS‐CoV‐2 infection. Controls had a negative result for SARS‐CoV‐2. Study samples were not tested for influenza, but there was no evidence of influenza circulation during the enrollment period. Multivariable logistic regression models examined the association between receipt of 2019–2020 influenza vaccine and PCR‐confirmed COVID‐19 with adjustment for potential confounders. Age as a spline and sample collection interval (days) were included a priori in all models. We assessed sex, high‐risk condition (present or not present), and calendar time (month of onset) as potential confounders. Variables were included in the final model if they changed the influenza vaccine coefficient by ≥5%. Presence of a high‐risk condition was defined by specific ICD‐10 codes in the electronic health record from October 1, 2019, through the date of enrollment and was consistent with those used by the US Flu VE Network.^11^ The primary analysis included all ages. Secondary analyses generated separate models for children (age 0 to <18 years) and adults (age ≥18 years). Covariates for the age‐stratified models were assessed separately, and age was included as a categorical variable for children. The Marshfield Clinic Research Institute Institutional Review Board approved this study.

## 3 RESULTS

The analysis included 1736 symptomatic patients. The median age was 38 years, and 60% were female. The interval from symptom onset to sample collection was less than 5 days in 78%. Twenty‐eight percent were positive for SARS‐CoV‐2 by RT‐PCR, and 65% had received a 2019–2020 influenza vaccine (Table 1). The peak month for illness onset was July with 594 enrolled. Almost half of all COVID‐19 cases in the study occurred in September (*n* = 240, 49%).

**TABLE 1:** Characteristics of enrolled patients, June 1 to September 30, 2020

Characteristic	Total, *n* (%)*n* = 1736	SARS‐CoV‐2 negative, *n* (%)*n* = 1249	SARS‐CoV‐2 positive, *n* (%)*n* = 487
Median age (IQR)	38 (19–57)	35 (17–55)	47 (29–61)
Age category			
≤8 years	189 (11)	178 (14)	11 (2)
9–17 years	191 (11)	144 (12)	47 (10)
18–49 years	740 (43)	532 (43)	208 (43)
50–64 years	394 (23)	260 (21)	134 (28)
≥65 years	222 (13)	135 (11)	87 (18)
Female	1034 (60)	752 (60)	282 (28)
Sample collection interval (onset to swab)			
0–2 days	817 (47)	599 (48)	218 (45)
3–4 days	541 (31)	393 (31)	148 (30)
5–7 days	291 (17)	201 (16)	90 (18)
8–10 days	87 (5)	56 (4)	31 (6)
Receipt of 2019–20 influenza vaccine			
No	606 (35)	423 (34)	183 (38)
Yes	1130 (65)	826 (66)	304 (62)
Onset month			
June	290 (17)	257 (21)	33 (7)
July	594 (34)	480 (38)	114 (23)
Aug	440 (25)	340 (27)	100 (21)
Sept	412 (24)	172 (14)	240 (49)
High‐risk condition	860 (50)	621 (50)	239 (49)

A similar proportion of SARS‐CoV‐2 cases and controls were vaccinated with 2019–2020 influenza vaccine (62% and 66%, respectively) (Table 2). Vaccination among children was comparable between SARS‐CoV‐2 cases and controls but slightly higher (66% and 71%, respectively). The adjusted odds ratio (aOR) for influenza vaccine receipt among SARS‐CoV‐2 cases was 0.80 (95% confidence Interval [CI], 0.62–1.03) (Table 2). Results were similar in age‐stratified analyses. AOR among adults was 0.83 (95% CI, 0.63–1.10) and 0.92 (95% CI, 0.47 to 1.80) among children.

**TABLE 2:** Multivariable analysis of 2019–2020 influenza vaccine receipt and symptomatic SARS‐CoV‐2 infection, June 1 to September 30, 2020

	Test‐positive SARS‐CoV‐2, *n* (%)	Test‐negative controls, *n* (%)	Unadjusted OR (95% CI)	Adjusted OR (95% CI)^a^	*p* value
All ages (*n* = 1736)					
Vaccinated	304 (62)	826 (66)	0.85 (0.68, 1.06)	0.80 (0.62, 1.03)	.1
Unvaccinated	183 (38)	423 (34)	Reference	Reference	
Adults age ≥18 years (*n* = 1356)					
Vaccinated	266 (62)	599 (65)	0.89 (0.71, 1.13)	0.83 (0.63, 1.10)	.2
Unvaccinated	163 (38)	328 (35)	Reference	Reference	
Children age <18 years (*n* = 380)					
Vaccinated	38 (66)	227 (71)	0.80 (0.44, 1.44)	0.92 (0.47, 1.80)	.8
Unvaccinated	20 (34)	95 (30)	Reference	Reference	

^a^ All ages and adult model adjusted for age (spline), sample collection interval, sex, high‐risk condition, and month of onset. Children model adjusted for age (0–8 years and ≥9 years), sample collection interval, sex, and month of onset.

## 4 DISCUSSION

In this TND analysis, we found no positive association between receipt of 2019–2020 seasonal influenza vaccine and laboratory‐confirmed, symptomatic SARS‐CoV‐2 infection. Findings were similar across adults and children. The results are consistent with other studies assessing influenza vaccine and coronavirus infection risk. Our results align with a TND study at primary care sites in Europe which reported an adjusted odds ratio of 0.93 (95% CI 0.66–1.32) for influenza vaccination in COVID‐19 cases versus test‐negative controls.^9^ Another TND study of data from Canadian primary care sites across seven influenza seasons prior to the pandemic reported an adjusted odds ratio of 1.04 (95% CI, 0.85–1.28) for influenza vaccination in seasonal coronavirus cases versus test‐negative controls.^3^ Vaccine did not affect seasonal coronavirus risk in children less than 20 years old (aOR 0.74, 95% CI 0.44–1.32) nor adults (aOR 1.11, 95% CI 0.89–1.38) in stratified analyses, which mirrors the findings in our study of SARS‐CoV‐2.

Multiple studies have now generated strong and consistent evidence that influenza vaccination does not increase the risk of COVID‐19. However, several of these studies have observed a modestly reduced risk of SARS‐CoV‐2 infection among individuals who received influenza vaccine in the prior one to two seasons. Two cohort studies reported a significantly reduced odds of a positive SARS‐CoV‐2 test in vaccinated versus unvaccinated individuals; the adjusted odds ratios were 0.76 (95% CI, 0.68–0.86) and 0.79 (95% CI 0.67–0.98) for vaccination in the 2019–2020 season.^7^, ^12^ The evidence for a true protective effect is inconclusive, and current understanding of the adaptive immune response to influenza vaccine does not provide a biological basis for heterologous protection. Residual confounding is another plausible explanation for the observed negative association between influenza vaccination and COVID‐19. The reduced risk in some studies could reflect differences in behavior between vaccinated and unvaccinated individuals. For example, receipt of influenza vaccination may also be associated with better adherence to protective measures against SARS‐CoV‐2 infection, including social distancing and masking. This possibility is consistent with an exploratory analysis that used a propensity score approach to examine the association between multiple different vaccines and SARS‐CoV‐2 infection.^13^ Several vaccines, including varicella, polio, measles‐mumps‐rubella (MMR), and pneumococcal conjugate were all associated with reduced risk of SARS‐CoV‐2 infection.

This study's strengths include prospective enrollment, confirmation of SARS‐CoV‐2 infection by RT‐PCR, and access to comprehensive electronic health records. Participants were systematically screened and enrolled based on symptoms of SARS‐CoV‐2 infection, reducing potential bias due to indication for testing (e.g., screening asymptomatic patients prior to surgical procedures). We assessed 2019–2020 influenza vaccination status using a previously validated vaccination registry to ensure accurate capture of vaccination status.^10^ We also conducted secondary, age‐stratified analyses, confirming children had similar findings to adults, with no observed impact of influenza vaccination on risk of SARS‐CoV‐2 infection. Influenza infections are a potential source of confounding in a test‐negative analysis of SARS‐CoV‐2 infection, but this study was conducted during the summer when influenza was not circulating. The timing of the study is a potential limitation. It was conducted 6–9 months after most influenza vaccines were administered. As a result, we were unable to assess short‐term effects of influenza vaccination on risk of SARS‐CoV‐2 infection.

In conclusion, this study did not identify any association between influenza vaccination and symptomatic SARS‐CoV‐2 infection in adults or children. The results from this study and others support current public health recommendations for annual influenza vaccination.

## CONFLICT OF INTEREST

HQM receives research funding from Seqirus unrelated to the present work. JPK and EAB have no conflicts to report.

## AUTHOR CONTRIBUTIONS

**Jennifer King:** conceptualization (equal); writing—original draft (lead); formal analyses (lead); writing—review and editing (equal). **Huong McLean:** conceptualization (equal); writing—review and editing (equal). **Edward Belongia:** conceptualization (equal); supervision (lead); writing—original draft (supporting); writing—review and editing (equal).

### PEER REVIEW

The peer review history for this article is available at https://publons.com/publon/10.1111/irv.12880.

## Data Availability

The data that support the findings of this study are not publicly available due to privacy or ethical restrictions. The data may be made available upon request.
